# Supplementary material for: Generation and Molecular Characterization of CRISPR/Cas9-Induced Mutations in 63 Immunity-Associated Genes in Tomato Reveals Specificity and a Range of Gene Modifications
Source: Front Plant Sci. 2020 Feb 4;11:10. doi: 10.3389/fpls.2020.00010 (PMC7010635; doi:10.3389/fpls.2020.00010)
Supplement: Supplementary file 1 [file DataSheet_1.zip › Data Sheet/Sup Figures S1_2_3_4.pdf]

A

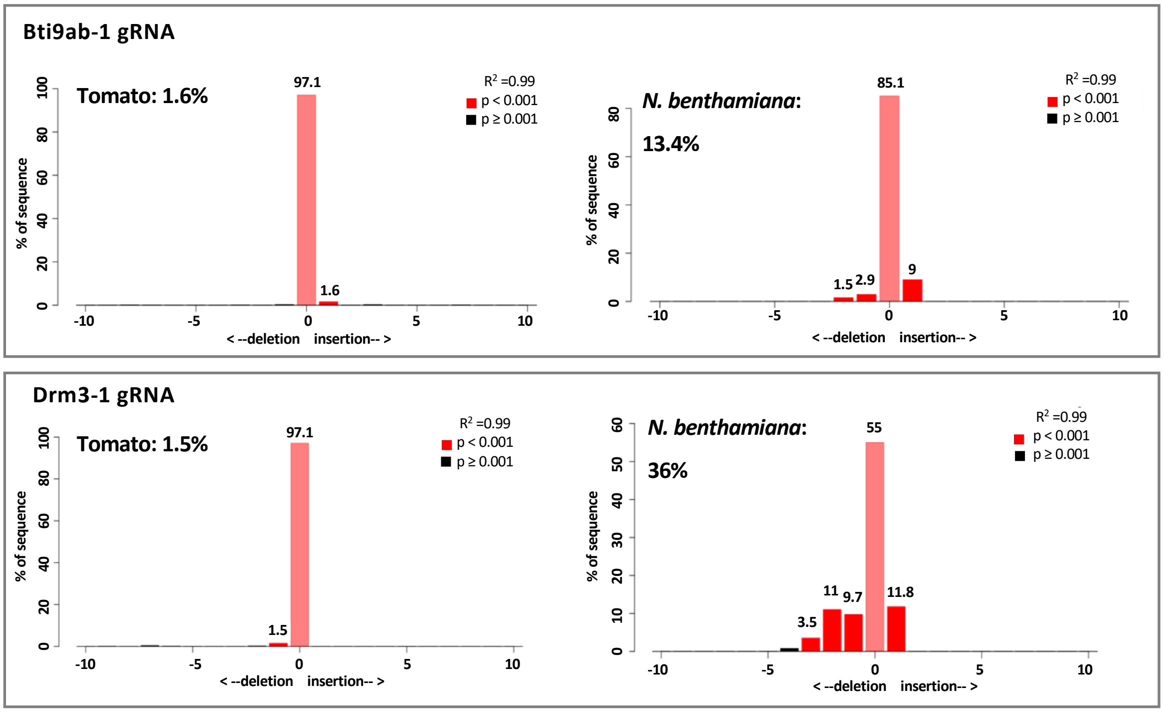

B

	gRNAs	Mutation efficiency	Protospacer + PAM [a]	Gene name	Gene ID
1	NbMai1-1	4.8%	GGTCGCATCTCATGTAATGTTGG	NbMai1 homolog1	Niben101Scf01157
2	NbMai1-1	0%	GGTCGCATCTCATGTAATGTTGG	NbMai1 homolog2	Niben101Scf04518
3	NbMai1-2	17.4%	GCTGCTTGCAGTATGAACCCAGG	NbMai1 homolog1	Niben101Scf01157
4	NbMai1-2	23.3%	GCTGCTTGCAGTATGAACCCAGG	NbMai1 homolog2	Niben101Scf04518
5	NbMai1-3	33.6%	GATGCTTGCAGTACGAACCTAGG	NbMai1 homolog3	Niben101Scf00837
6	NbMai1-3	36.6%	GATGCTTGCAGTACGAACCTAGG	NbMai1 homolog4	Niben101Scf02175
7	NbMai1-4	24.2%	GCTCGGAATTCCTAAGAATGAGG	NbMai1 homolog3	Niben101Scf00837
8	NbMai1-4	26.9%	GCTCGGAATTCCTAAGAATGAGG	NbMai1 homolog4	Niben101Scf02175
9	Drm3-1	36.0%	GCCACTATCTGGCCGGGGACCGG	NbDrm3	Niben101Scf01041Ctg024
10	Bti9ab-1	13.4%	GATTCCGGTCGCCTGGCTGATGG	NbBti9	Niben101Scf01037

C

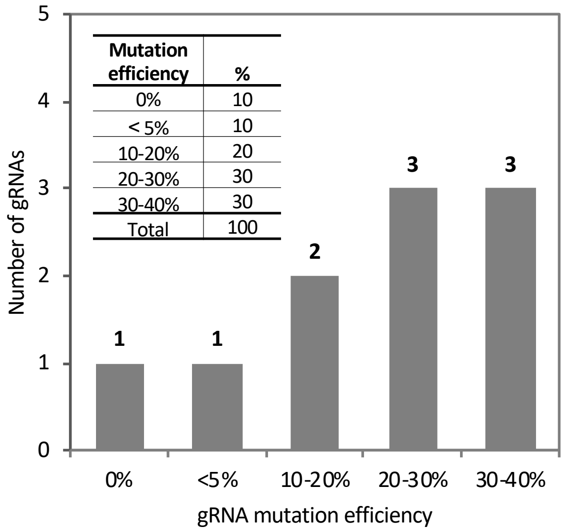

Figure S1. Evaluation of gRNA efficiency by agroinfiltration in *Nicotiana benthamiana* leaves. Related to Figure 1. (**A**) Comparison of the efficiency of the same gRNA to create mutations in tomato or *N. benthamiana*. Agroinfiltration and mutation analysis were the same as described in Fig. 1. **(B)** Efficiency of 10 gRNAs in *N. benthamiana* by agroinfiltration. [a] PAM (NGG) are underlined. **(C)** Distribution of gRNA mutation frequency in *N. benthamiana*. Inset on the top left shows the percentage of each mutation frequency range.

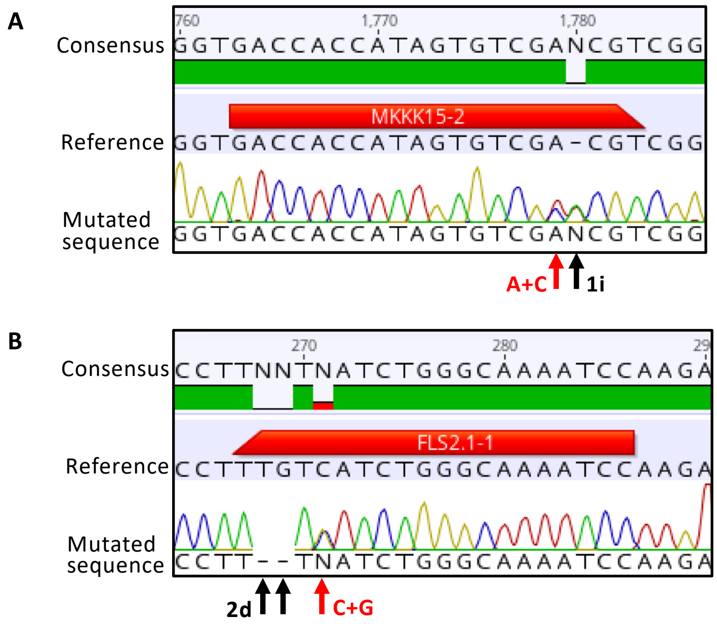

Figure S2. Base substitutions in tomato mutant plants by CRISPR/Cas9. (**A**) Mutation caused by the MKKK15-2 gRNA was 1-bp insertion plus a “A to C” substitution in one copy of the allele. 1i: 1-bp insertion. (**B**) Mutation caused by the FLS2.1-1 gRNA was 2-bp deletion plus a “C to G” substitution in one copy of the allele. 2d: 2-bp deletion.

A

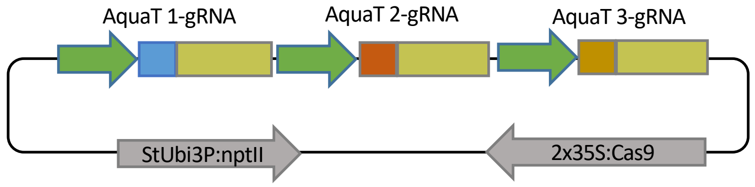

B

Targeted genes	# of mutants for each target gene	# of plants genotyped	# of plants with edits	Number of plants with mutations in one, two, or three genes		
				one	two	three
Aquaporin Transporter 1/2/3	AquaT 1: **10**; AquaT 2: **6**; AquaT 3: **4**	12	10	3	4	3

Figure S3. Multiplex genome editing by CRISPR/Cas9 in tomato. (**A**) Schematic shows the order of three gRNAs cassettes targeting *Aquaporin Transporter* genes in the p201N:Cas9 vector. AquaT: Aquaporin Transporter. **(B)** Summary of multiplex editing of three AquaT genes in tomato.

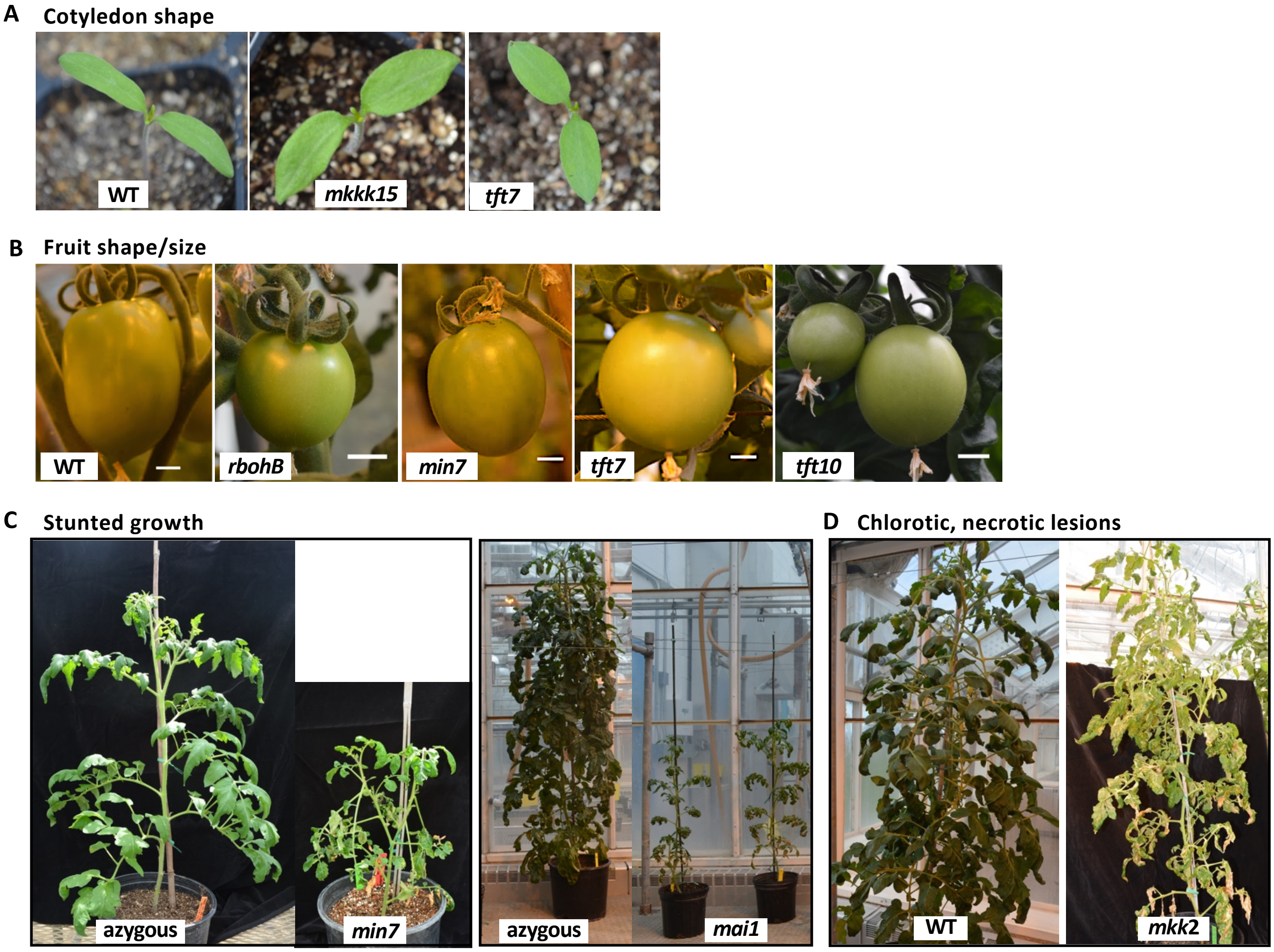

Figure S4. Morphology defects in some CRISPR-induced tomato mutants knocking out immunity-associated genes. (**A**) Changed cotyledon shape from elongate (wild-type, WT) to ovate (*mkkk15/tft7* mutants). Cotyledon size is similar between WT and mutant plants. (**B**) Change in fruit shape and size in some of the mutant plants. Scale bar: 1 cm. (**C**) Some mutants show stunted growth. Azygous plants are from the same transformation event but contain two copies of the wild-type alleles. Azygous and mutant plants are the same age. (**D**) The mkk2 mutant showed chlorotic and necrotic lesions in leaves.
