# Supplementary material for: Generation and Molecular Characterization of CRISPR/Cas9-Induced Mutations in 63 Immunity-Associated Genes in Tomato Reveals Specificity and a Range of Gene Modifications
Source: Front Plant Sci. 2020 Feb 4;11:10. doi: 10.3389/fpls.2020.00010 (PMC7010635; doi:10.3389/fpls.2020.00010)
Supplement: Supplementary file 1 [file DataSheet_1.zip › Data Sheet/Sup Tables S4_5_6_7.pdf]

Table S4. Tomato mutant events selected for off-target analysis showed morphology defects. Related to Table 3.

Mutant plants tested for off-targets	Aberrant morphology	gRNAs that induced mutations	gRNA sequence
SOBIR-E1	No meristem in most T1 seedlings	SOBIR	GTTTATAGAGCAGAGCTACC
Min7-E4	Stunted growth; sensitive to abiotic stress	Min7-2	GAACAGGGTCTGTGGCTGTG
Min7-E5	Stunted growth; sensitive to abiotic stress	Min7-2	GAACAGGGTCTGTGGCTGTG
Min7-E6	Delayed/aborted flowers; few fruit set	Min7-2	GAACAGGGTCTGTGGCTGTG
RALF1-E4	Longer internodes	RALF1.1-2	GAAGCTCCTCTCCTTGAACA
RALF2-E1	Etiolated seedlings	RALF1.2-2	gGTTGTAGTAGGAAGCTCCA
PBL-T1-E1	Aborted flowers	PBL-T1-2	GATGGCGAGTCTGGTCAAAT
Mai5/PBL-T1-E5	Delayed or aborted flowering/few fruit set	Mai5-3	gTAGATCGTAATGGATTGCA
Mai5/PBL-T1-E7	Delayed or aborted flowering/few fruit set	Mai5-2	GTCTACGAATATATGCCATT
MKKK15-E2	Round fruits; oval cotyledons	MKKK15-2	GACCACCATAGTGTCGACGT
TFT1/TFT7-E1	Round fruits (wild-type is oval)	TFT1-2	GTTCACTTCGAGCAGCGTGG
TFT1/TFT7-E1	Round fruits (wild-type is oval)	TFT7-2	GAATGTAATCGGAGCAAGAA
TFT10-E1	Aborted flowers; Round fruits	TFT10-1	GATTCACTGGTAGTAGCAGA
Cathepsin B1-E5	Round cotyledon; fruit shape changed	Cathepsin B1-1	GGCCTCAGTGTAGCACTATT
ERF5-E2	Delayed or aborted flowering/few fruit set	ERF5-1	GGTATTGCTACGTTCAGAGA
ERF5-E3	Delayed flowering	ERF5-1	GGTATTGCTACGTTCAGAGA
MKK2-E1	Delayed/aborted flowering/few fruit set	MKK2-2	GAAGACGGACAGAATCCTCG
MKK2-E2	Elongated fruits (wild-type is oval)	MKK2-2	GAAGACGGACAGAATCCTCG

Table S5. Transient genome editing by CRISPR/Cas9 in tomato stable transformation. Related to Figure 3.

Plants	Target gene	Gene Solyc#	Mutations	gRNAs that induced mutations	gRNAs detected in the plants[a]
1	AOX	Solyc08g075550	-1 bp/WT	AOX-2	WRKY11-4
	AOX	Solyc08g075550	+1 bp/WT	AOX-1	WRKY11-4
	RALF2	Solyc01g099520	-2 bp/WT	RALF1.2-1	WRKY11-4
2	ERF5	Solyc03g093560	+1 bp/-11 bp/WT	ERF5-1	WRKY11-4
3	MAP3Ka	Solyc11g006000	+1 bp/+50 bp	MAP3Ka-3	MKKK66-1 and MKK1-1
4	MKKK15	Solyc02g065110	-5 bp/WT	MKKK15-2	MKKK20-1
5	MKKK15	Solyc02g065110	-5 bp/WT	MKKK15-2	MKKK20-1
6	MKKK15	Solyc02g065110	+1 bp/WT	MKKK15-2	PCR failed
7	PBL-T1	Solyc09g007170	+1 bp/-5 bp	PBL-T1-1	Both Mai5/PBL-T1
8	RALF2	Solyc01g099520	+1 bp/WT	RALF1.2-1	AOX-1

[a] These gRNAs were pooled together with other gRNAs for one transformation experiment, detected in transgenic plants by PCR and sequencing, but did not induce mutations at the target site.

Table S6. Primers used for cloning and genotyping.

Primer name	Primer sequence	Purpose
Stubi3P218R	ACATGCACCTAATTTCACTAGATGT	For gRNA detection in transgenic plants
MtU6252F	GCATCCCAGTAGGTGAAAGTCGAG	For gRNA detection in transgenic plants
p201R	CGCGCCGAATTCTAGTGATCG	For gRNA detection in transgenic plants
nosT-rev2	CAAGACCGGCAACAGGATTCAATC	For Cas9 detection in transgenic plants
Cas9F7	GGGTCTCCCGAAGATAATGAGC	For Cas9 detection in transgenic plants
SwaI_MtU6F	GATATTAATCTCTTCGATGAAATTTATGCCTATCTTATATGATCAATGAGG	For generation of the triple-gRNA construct
SpeI_Scaffold_R	GTCATGAATTGTAATACGACTCAAAAAAAAGCACCGACTCGGTG	For generation of the triple-gRNA construct
UNS1_MtU6F	CATTACTCGCATCCATTCTCATGCCTATCTTATATGATCAATGAGG	For generation of the triple-gRNA construct
UNS1_Scaffold_R	GAGAATGGATGCGAGTAATGAAAAAAAGCACCGACTCGGTG	For generation of the triple-gRNA construct
UNS2_MtU6F	GCTGGGAGTTCGTAGACGGAATGCCTATCTTATATGATCAATGAGG	For generation of the triple-gRNA construct
UNS2_Scaffold_R	TCCGTCTACGAACTCCCAGCAAAAAAAGCACCGACTCGGTG	For generation of the triple-gRNA construct

Table S7. Primers used for off-target analysis (related to Table 3).

gRNA tested	Primer name	Forward primer	Reverse primer
CathepsinB1-1	B1-offtarget1	GTTTGCCTTATTACATGGTCTTGG	GTCAGGACTCAAACTCGAAATC
	B1-offtarget2	CGAAAAAAGCCTTGACACATAAC	GATTTGATGGTCTCACGATGAC
ERF5-1	ERF5-offtarget1	GATGCTTCGTTTCTCCACCTG	CAATTCGACGGTGTTAATGGTG
	ERF5-offtarget2	GAAGCCTCCTAAGAAACCTCATC	CTCATCATTTTGTTGCTTAAAGTTTGC
Fen-1	Fen-offtarget1	GAAGACTACTCAACTCTATGTGTC	GTTGGGAGTAGTATTCAGCACTG
Mai5-2	Mai5-offtarget1	GAAGACTACTCAACTCTATGTGTC	GTTGGGAGTAGTATTCAGCACTG
Mai5-3	Mai5-offtarget2	GTTTGTACCTGGACAGATTGTG	GATTGGTCGTTTCGTGTAGATAC
	Mai5-offtarget3	GAAGCCTCAACTTGCACGGA	CCACTTCCATAGCCCATAAGAG
	Mai5-offtarget4	CTTCGAATCATAGACCTCGCTAG	GCTTTCTTGTTCCAACTCTGTCA
MKK2-1	MKK2-offtarget1	GTTAGATTCTTAGGCAATTCACTGG	CAGTCTCATTCTCAGGATTCATC
MKKK15-2	MKKK15-offtarget1	GCTTGTAAGAGTCCCTTGAGAAG	GCAAGACGAATAGGGATATACTAAG
PBL-T1-2	PBLT1-offtarget1	CATAAGTTGAGGGTGGCGAATG	CCAGCGGCTTATAGAATTTCGG
RALF2-2	RALF2-offtarget1	CCTAAAGTGAACTACGCATGAATTC	CGTGTAGCCTGATATGCAAAGG
SOBIR	SOBIR-offtarget1	GTGGCTTAGTTGAATCCCGAAG	CTACTGTTGTTATCTTGACCGATC
TFT10-1	TFT10-offtarget1	GCAGTAGAGTAGTTGAAGTCATC	GTGGACATAACTCGATACTTTTC
	TFT10-offtarget2	GTGTGCGAGAAACAGTAGCTG	GCAGTAACATCAGAATCCACAGC
TFT7-2	TFT7-offtarget1	GCAGATAATGATTGTGGTCACG	CATGATGCCTTTTAAGAGACTTTTG
	TFT7-offtarget2	CTCAAATCTCATCCAAATCATGTCC	CTGCTGATATAGTGAACTGGTAAC
